# AMP-activated protein kinase can be allosterically activated by ADP but AMP remains the key activating ligand

**DOI:** 10.1042/BCJ20240082

**Published:** 2024-04-22

**Authors:** Simon A. Hawley, Fiona M. Russell, D. Grahame Hardie

**Affiliations:** Division of Cell Signalling and Immunology, School of Life Sciences, University of Dundee, Dundee DD1 5EH, Scotland, U.K.

**Keywords:** adenine nucleotides, ADP, AMP, AMPK, CBS repeats, LKB1

## Abstract

The AMP-activated protein kinase (AMPK) is a sensor of cellular energy status. When activated by increases in ADP:ATP and/or AMP:ATP ratios (signalling energy deficit), AMPK acts to restore energy balance. Binding of AMP to one or more of three CBS repeats (CBS1, CBS3, CBS4) on the AMPK-γ subunit activates the kinase complex by three complementary mechanisms: (i) promoting α-subunit Thr172 phosphorylation by the upstream kinase LKB1; (ii) protecting against Thr172 dephosphorylation; (iii) allosteric activation. Surprisingly, binding of ADP has been reported to mimic the first two effects, but not the third. We now show that at physiologically relevant concentrations of Mg.ATP^2−^ (above those used in the standard assay) ADP binding does cause allosteric activation. However, ADP causes only a modest activation because (unlike AMP), at concentrations just above those where activation becomes evident, ADP starts to cause competitive inhibition at the catalytic site. Our results cast doubt on the physiological relevance of the effects of ADP and suggest that AMP is the primary activator *in vivo*. We have also made mutations to hydrophobic residues involved in binding adenine nucleotides at each of the three γ subunit CBS repeats of the human α2β2γ1 complex and examined their effects on regulation by AMP and ADP. Mutation of the CBS3 site has the largest effects on all three mechanisms of AMP activation, especially at lower ATP concentrations, while mutation of CBS4 reduces the sensitivity to AMP. All three sites appear to be required for allosteric activation by ADP.

## Introduction

The AMP-activated protein kinase (AMPK) is a sensor of cellular energy status that also responds to changes in availability of nutrients such as glucose and fatty acids, as well as to certain types of cellular damage [1,2]. AMPK is expressed in essentially all eukaryotic cells as heterotrimeric complexes comprising catalytic α subunits and regulatory β and γ subunits, each of which occur in mammals as multiple isoforms (α1, α2, β1, β2, γ1, γ2, γ3) encoded by distinct genes [3]. In response to energy imbalance, signalled by increases in cellular AMP relative to ATP, binding of AMP causes AMPK activation by three complementary mechanisms [4]: (i) promotion of phosphorylation of Thr172 on the AMPK-α subunit by the upstream kinase LKB1; (ii) protection against dephosphorylation of Thr172 by protein phosphatases; (iii) allosteric activation.

If the adenylate kinase reaction (2ADP ↔ ATP + AMP) is close to equilibrium, which appears to be the case in many eukaryotic cells, the AMP:ATP ratio will vary as the square of the ADP:ATP ratio [5], making the former potentially a more sensitive indicator of energy stress than the latter. Despite this, there has been some controversy within the AMPK field as to whether AMP or ADP is the principal activating signal. In particular, it has been reported that binding of ADP mimics two of the three effects of AMP, i.e. enhanced phosphorylation [6] and inhibition of dephosphorylation [7] of Thr172. We have confirmed these findings, although we find that both effects require concentrations of ADP that are typically 10-fold higher than those of AMP [8].

Adenine nucleotides regulate AMPK by binding to the sites formed by the four tandem CBS repeats located on the γ subunit [9]. These sequence motifs of ≈60 residues are also found as tandem repeats in many other proteins, including cystathionine β-synthase (from which the acronym CBS derives). Single pairs of CBS repeats form pseudo-dimers that generate, in the intervening cleft, either one or two binding sites for regulatory ligands that usually containing adenosine (e.g. AMP, ATP, S-adenosyl methionine [9,10]). In AMPK-γ subunits the four CBS repeats form two pseudo-dimers arranged head-to-head [11], creating a disk-like structure with four potential ligand-binding sites located close together in the centre. One site (CBS2) appears to be always unoccupied; the remaining three bind adenine nucleotides with their adenine groups facing away from each other and interacting with hydrophobic residues specific to each CBS repeat, while their phosphate groups face towards each other and bind conserved histidine and arginine side chains that may be derived either from the same or a neighbouring repeat [11]. There is general agreement that CBS4 (originally termed site 3 [11]) binds AMP tightly in a non-exchangeable manner [11,12], leaving CBS1 and CBS3 as the sites where adenine nucleotides compete for binding. Consistent with this, estimates of dissociation constants for various bacterially expressed heterotrimers, either by surface plasmon resonance [13] or by displacement of fluorescent nucleotide analogues [7,14], were compatible with two AMP-binding sites: one of high (*K*_d_ = 2–4 µM) and one of low (*K*_d_ = 40–400 µM) affinity. While it was originally proposed that CBS1 was the high-affinity site [7], more recent evidence suggests that CBS3 is the high-affinity site [14]. When measured in the presence of Mg^2+^ and the kinase inhibitor staurosporine (the latter blocking binding of nucleotides to the catalytic site on the α subunit) the estimated *K*_d_ values for ADP (12–24 µM) and ATP (100–500 µM) at the CBS3 site were one and two orders of magnitude higher, respectively, than that of AMP (1–2 µM) [13]. Thus, the CBS3 site is able to sense changes in AMP despite the presence of much higher concentrations of ADP and ATP. Consistent with a key regulatory role for this site, when AMP binds to CBS3 the bound nucleotide interacts with the α-linker (also known as the α-subunit Regulatory Interaction Motif or α-RIM), a region of extended, flexible polypeptide that links the autoinhibitory and C-terminal domains of the α subunit [7,15]. The α-linker is thought to dissociate from the γ subunit when ATP displaces AMP at the CBS3 site [16], causing a major conformational change that may account for both allosteric inhibition and promotion of net dephosphorylation of Thr172 by ATP [17].

The binding of AMP at the CBS3 site might, therefore, be responsible for all three of its effects, i.e. (i) allosteric activation; (ii) promoting Thr172 phosphorylation, and (iii) inhibiting Thr172 dephosphorylation. This hypothesis is addressed further in the second part of this paper, but if it is correct, why would binding of ADP at the same site not have the same three effects as AMP? Although ADP binding has been reported to mimic effects (ii) [6,8] and (iii) [7,8], it has not been shown to cause allosteric activation. Under standard assay conditions of 5 mM Mg^2+^ and 200 µM ATP, Xiao et al. [7] reported that ADP caused no allosteric activation of bacterially expressed AMPK at any concentration up to 200 µM, while Oakhill et al. [6] reported that 200 µM ADP caused a small inhibition. In both cases, 200 µM AMP caused ∼2-fold allosteric activation under the same conditions. In this paper we have re-investigated the question of whether ADP causes allosteric activation of AMPK. We have also made mutations to key hydrophobic residues in the CBS1, CBS3 and CBS4 sites and have shown that binding of AMP at the CBS3 site is critical for all three activating effects of AMP, at least when assays are performed at lower ATP concentrations.

## Results

### Allosteric activation of rat liver AMPK by AMP and ADP

To study allosteric effects, we initially used a preparation of native rat liver AMPK [4]. While not homogeneous, this preparation has a high specific kinase activity (1.3 µmol/min/mg) and is a combination of the α1, α2, β1, β2 and γ1 subunit isoforms (Supplementary Figure S1). Being from a mammalian source both β subunit isoforms would be N-myristoylated [18] and we have shown previously that the preparation is not contaminated with any protein phosphatases that dephosphorylate Thr172 [4]. We assessed the potential allosteric effects of both AMP and ADP at three different concentrations of ATP. We maintained Mg^2+^ ions at a constant 4.8 mM excess above total ATP, a design that ensures that the concentration of the Mg.ATP^2−^ complex varies as a fixed proportion of total ATP [19].

**Figure 1.**
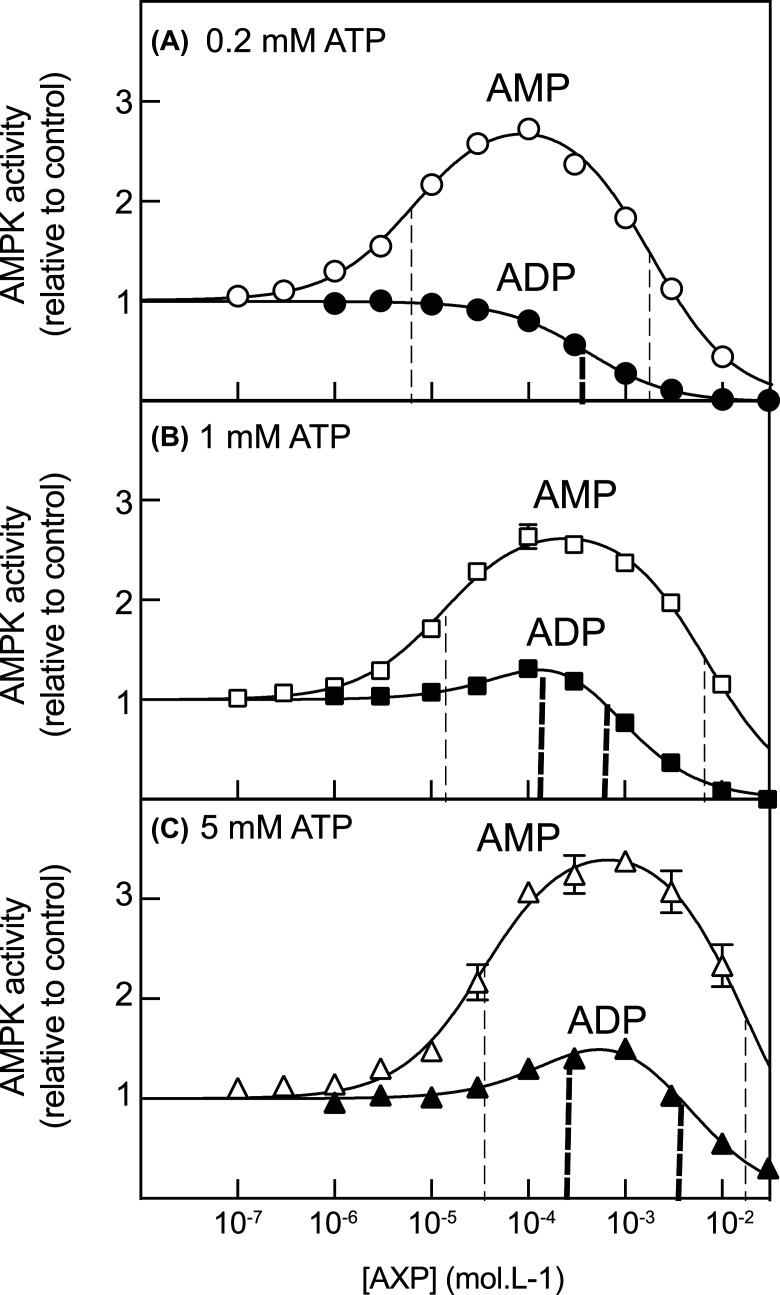
Allosteric activation of rat liver AMPK by AMP and ADP at three different concentrations of ATP. (**A**) 200 µM ATP; (**B**) 1 mM ATP; (**C**) 5 mM ATP. [Mg^2+^] was maintained at a constant excess of 4.8 mM above [ATP] as recommended previously [19]. Results (mean ± SD, *n* = 2) were fitted to the equation: *Y* = 1 + (((Activation-1) × *X*)/(EC_50_ + *X*))-(((activation) × *X*)/(IC_50_ + *X*)), where *Y* is the activity, *X* is the concentration of AMP or ADP, Activation is the extrapolated maximal activation by AMP or ADP, EC_50_ is the concentration of AMP or ADP causing half-maximal activation, and IC_50_ is the concentration of AMP or ADP causing half-maximal inhibition. The dashed vertical lines show the estimated EC_50_ and IC_50_ values, faint lines for AMP and bold lines for ADP. A reliable estimate of EC_50_ could not be computed for ADP at 0.2 mM ATP (graph (A)). Best-fit values of all parameters used to generate the curves are listed in Supplementary Table S1.

As previously reported [4], with AMP we obtained bell-shaped curves due to activation at low concentrations combined with inhibition at higher concentrations (Figure 1). We have previously shown that the allosteric activation is due to binding of AMP to one or more of the regulatory sites on the γ subunit [9], whereas the inhibition is due to binding of AMP at the catalytic site within the α subunit kinase domain [4]. Consistent with the fact that ATP competes with AMP at both sites, as the ATP concentration was progressively raised from 0.2 to 1.0 and 5 mM, the bell-shaped curves shifted to the right, towards higher concentrations of AMP. The data were fitted to an equation for activation at one site and inactivation at a second site, as detailed in the legend to Figure 1. The best-fit parameters are shown in Supplementary Table S1, and these values were used to draw the curves in Figure 1A–C; the best-fit estimates for EC_50_ and IC_50_ are indicated by dashed vertical lines, faint lines for AMP and heavier lines for ADP.

The results obtained with AMP were similar to those obtained previously [4], except that the degree of activation over basal (3- to 4-fold) was somewhat smaller. As the concentration of ATP was increased from 0.2 to 1 and then 5 mM, the EC_50_ for AMP (concentration giving half-maximal activation) increased from 6.4 to 14 to 36 µM. Similarly, the IC_50_ for AMP (concentration giving half-maximal inhibition) increased from 1.7 to 6.9 to 17 mM. However, at each ATP concentration the EC_50_ was 2–3 orders of magnitude lower than the IC_50_, so that the activating and inhibitory phases for AMP were well separated.

Interestingly, ADP also yielded some allosteric activation of AMPK (Figure 1), although much less than that obtained with AMP. In agreement with previous results [7] no activation was detectable at 0.2 mM ATP, but at 1 and 5 mM ATP significant activation above basal, which reached observed maximal extents of ≈25% and ≈50% respectively, was obtained. A major difference between the effects of AMP and ADP was that for AMP the IC_50_ values for inhibition were 2–3 orders of magnitude higher than the EC_50_ values for activation (faint dashed lines in Figure 1), but for ADP the IC_50_ and EC_50_ values (thicker dashed lines in Figure 1) were much closer to each other (6-fold difference at 1 mM ATP and 18-fold at 5 mM ATP). Because the concentrations of ADP causing activation were only slightly lower than those causing inhibition, the best-fit estimates for certain parameters for ADP activation were subject to some uncertainty (Supplementary Table S1). The best-fit values for maximal activation were 2.4-fold at 1 mM ATP and 2.0-fold at 5 mM ATP. However, these extrapolated extents of maximal activation were never actually attained, because ADP began to inhibit the kinase before maximal activation was reached.

### Allosteric activation by ADP is not due to generation of AMP in the assay

**Figure 2.**
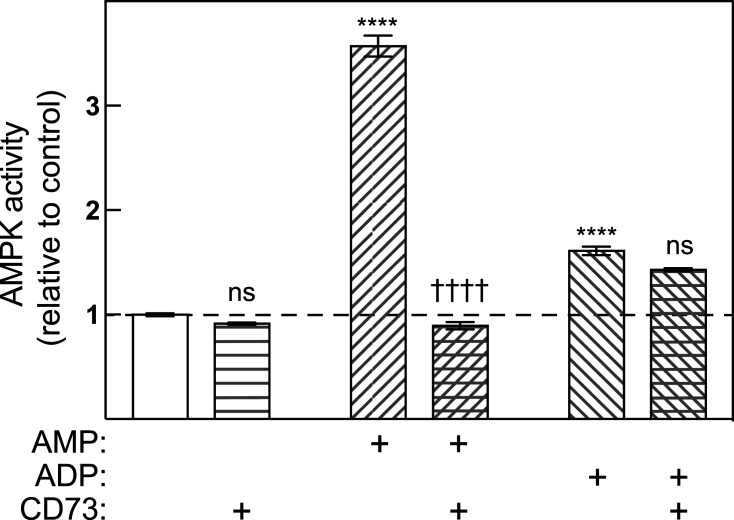
Allosteric activation of rat liver AMPK by AMP and ADP in the presence and absence of the nucleotidase CD73. Allosteric activation was assessed as in Figure 1 with a single fixed concentration of AMP or ADP (0.3 or 1 mM respectively) in the presence or absence of CD73 (550 ng). Statistical significance of differences in results (mean ± SEM, *n* = 3) were assessed by two-way ANOVA. Effect of nucleotide: ****P < 0.0001; effect of CD73: ^††††^*P* < 0.0001, ns, not significant.

To rule out the possibility that the allosteric activation of AMPK by ADP was due either to contamination of our ADP preparation by small amounts of AMP, or to generation of AMP from ADP during the assay (both of which can occur [4,20]), we studied allosteric activation of rat liver AMPK by both nucleotides in the presence or absence of CD73, a 5′-nucleotidase that converts AMP to adenosine but is completely inactive against ADP. The presence of CD73 in the assay did not significantly affect basal AMPK activity or the modest allosteric activation by ADP, but completely abolished the much larger allosteric activation by AMP (Figure 2).

### ADP is a competitive inhibitor with ATP at the catalytic site

**Figure 3.**
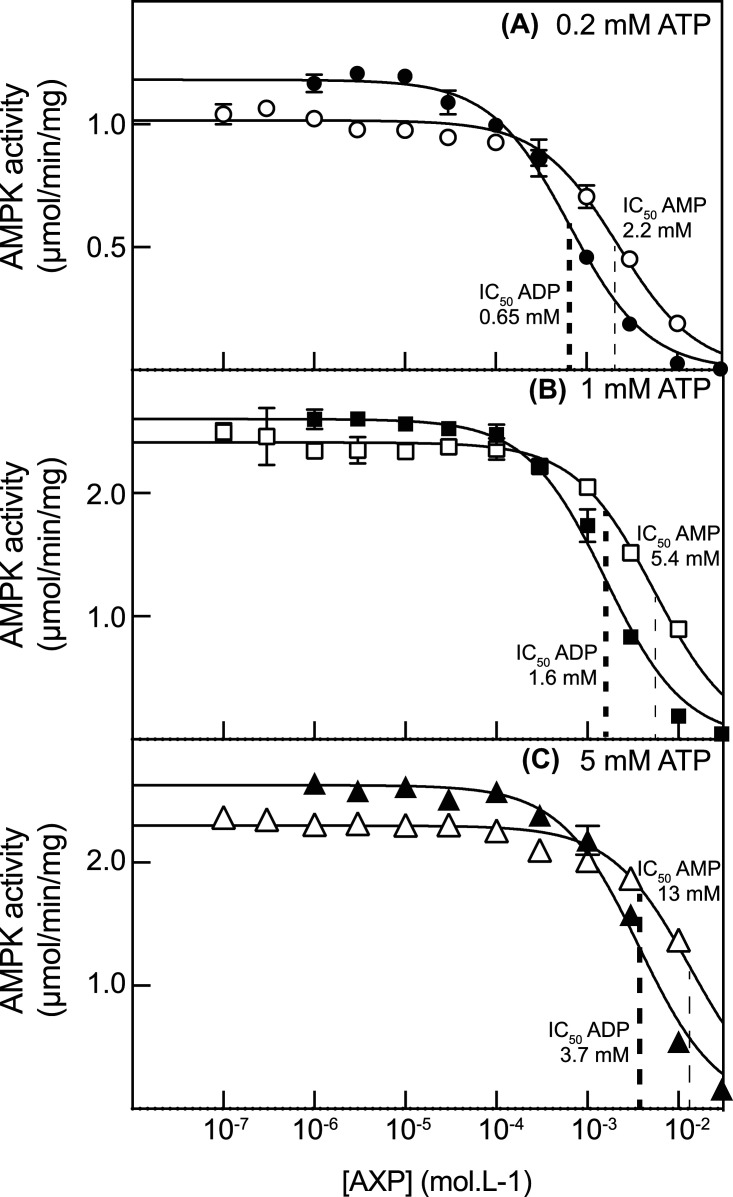
Inhibition of GST:α2-kinase domain fusion by AMP or ADP. The kinase domain (phosphorylated on Thr172 by the LKB1:STRADα:MO25α complex) was incubated with various concentrations of AMP or ADP as in Figure 1 using [ATP] at 0.2 (**A**), 1.0 (**B**) or 5.0 mM (**C**) and keeping [Mg^2+^] in a constant excess of 4.8 mM above [ATP] [19]. Results (mean ± SD, *n* = 2) were fitted to the equation: *Y* = Basal-(Basal × *X*)/(IC_50 _+ *X*) where *Y* is the kinase activity, *X* is the concentration of AMP or ADP, and IC_50_ is the concentration of AMP or ADP causing half-maximal inhibition. Dashed vertical lines show the estimates of IC_50_ values, and best-fit values of all parameters used to generate the curves are listed in Supplementary Table S1.

To confirm that the inhibition of kinase activity by ADP at high concentrations was due to competition with ATP at the catalytic site on the α subunit, we repeated the assays using a glutathione-*S*-transferase- (GST-) tagged human α2 kinase domain that had been phosphorylated by LKB1. The results (Figure 3) confirmed that ADP did not cause any activation of the isolated kinase domain but did inhibit it with IC_50_ values in the low mM range, which increased slightly as the ATP concentration was increased (bold dashed vertical lines in Figure 3 and Supplementary Table S1), consistent with ADP and ATP being in competition (Figure 3). These IC_50_ values were similar to those obtained with the intact heterotrimers from rat liver (Supplementary Table S1). AMP also inhibited the GST-α2 kinase domain construct, albeit with IC_50_ values (faint dashed lines in Figure 3) ∼3-fold higher than ADP. In other words, AMP is a less potent inhibitor at the catalytic site than ADP.

### Allosteric activation of human recombinant α2β2γ1 complex by AMP and ADP

To study allosteric activation with a more well-defined AMPK complex, and to study the effects of mutations in individual adenine nucleotide-binding sites on the γ subunit, we expressed a human α2β2γ1 complex in *Escherichia coli* and purified it utilizing the polyhistidine tag at the N-terminus of the α2 subunit. After Thr172 phosphorylation, the wild type (WT) complex had a slightly higher specific kinase activity (2.6 µmol/min/mg protein) than the rat liver preparation, and was an almost pure α2β2γ1 complex as judged by Coomassie Blue staining and Western blotting after SDS:PAGE (Supplementary Figure S1). However, it had not been co-expressed with an N-myristoyl transferase so would not have been N-myristoylated on the β2 subunit. The complex was expressed not only with the WT γ1 sequence but also with mutations affecting hydrophobic residues previously shown to be involved in binding of the adenine moieties of nucleotides at CBS1 (L129D/V130D), CBS3 (V276G/L277G) and CBS4 (I312G) [12,14]. All four complexes co-purified as heterotrimeric 1:1:1 complexes as expected, and were phosphorylated and activated equally well either by CaMKK2 or the LKB1: STRADα:MO25α complex (Supplementary Figure S2). For the studies described below we phosphorylated Thr172 using a GST-tagged CaMKK2 and repurified AMPK using size exclusion chromatography and a glutathione-Sepharose column, which remove CaMKK2.

**Figure 4.**
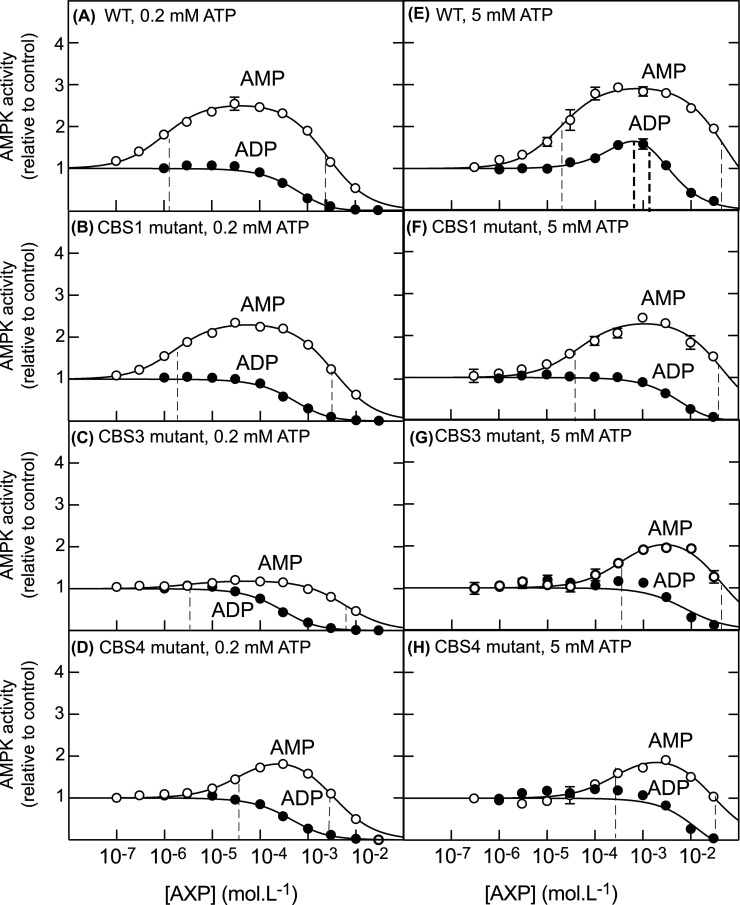
Allosteric activation of WT and mutant human α2β2γ1 AMPK complex by AMP and ADP at three different concentrations of ATP. The α2β2γ1 complex was phosphorylated on Thr172 prior to assay using CaMKK2. The γ1 subunit was either (A/E): WT; (B/F): a CBS1 mutant (L129D/V130D); (C/G): a CBS3 mutant (V276G/L277G); (D/H): a CBS4 mutant (I312G). Results are means of duplicate assays ± SD; error bars are not shown if they were smaller than the symbols used for mean values. Assays and curve fitting were as in Figure 1 at either 0.2 mM (left panels) or 5 mM (right panels) ATP. Best-fit values for all parameters used to generate the curves are listed in Supplementary Table S1.

The WT human complex yielded results remarkably similar to those obtained with the rat liver preparation. Using low ATP in the assay (0.2 mM) AMP activated the WT α2β2γ1 complex up to 2.6-fold at concentrations between 0.1 and 100 µM, and then inhibited at higher concentrations within the mM range (Figure 4A). The L125D/V130D mutations in CBS1 did not significantly alter either of these effects, while the V276G/L277G mutations in CBS3 almost completely abolished the activating effect at low AMP, with just 20% activation over basal remaining (Figure 4A–C). Interestingly, the I312G mutation in the CBS4 site only marginally reduced maximal activation (from 2.6-fold in the WT to 2.1-fold), but it increased the EC_50_ for activation by 40-fold, from 0.99 to 38 µM (Figure 4D). Using this low concentration of ATP in the assay, ADP did not cause allosteric activation either with the WT complex or any of the mutants, although the inhibition by mM concentrations of ADP, which we show above is due to competition with ATP at the catalytic site, was still evident (Figure 4A–D).

When the assays were conducted at a higher, more physiological concentration of ATP (5 mM), ADP now allosterically activated the WT human α2β2γ1 complex, similar to the native rat liver AMPK (Figure 4). The extrapolated maximal activation by ADP was 9.9-fold, although the maximal activation actually reached was only 1.6-fold, because the EC_50_ for activation and the IC_50_ for inhibition were very close together (0.78 and 1.2 mM respectively), so that inhibition occurred before maximal activation had been reached. Maximal allosteric activation by AMP with the WT complex increased marginally from 2.6-fold at 0.2 mM ATP to 3.0-fold at 5 mM ATP. The values for EC_50_ and IC_50_ were separated by more than three orders of magnitude (19 µM and 52 mM), so that activation was essentially complete before inhibition became evident.

Surprisingly, when the assays were conducted at 5 mM ATP, the CBS1 site appeared to become more important. Thus, mutation of hydrophobic residues in this site did not affect the EC_50_ for AMP although marginally reducing the maximal allosteric activation (from 3.0- to 2.4-fold), but it abolished allosteric activation by ADP. Conversely, mutation of the CBS3 or CBS4 sites increased the EC_50_ for AMP by at least two orders of magnitude but did not abolish allosteric activation completely. With these mutants, any remaining allosteric activation by ADP was too small to allow accurate estimates of EC_50_ or maximal activation.

### The CBS3 site is crucial for all three activating effects of AMP on AMPK

**Figure 5.**
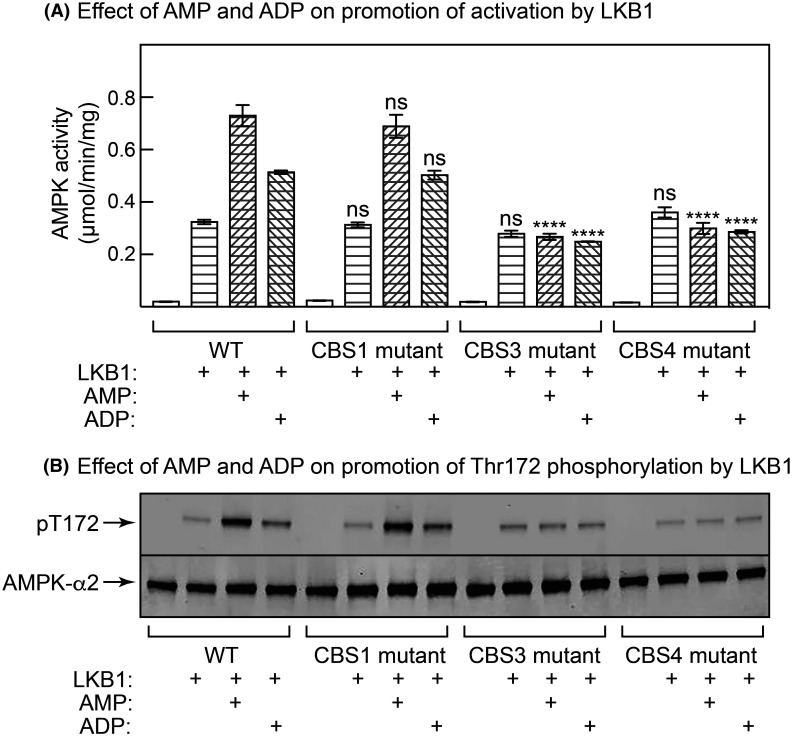
Effects of AMP or ADP on AMPK activation and Thr172 phosphorylation by the LKB1:STRADα:MO25α complex. Unphosphorylated human α2β2γ1 complexes (WT, or with L129D/V130D, V276G/L277G or I312G mutations affecting CBS1, CBS3 or CBS4 respectively) were incubated with ATP and a fixed concentration of the LKB1:STRADα:MO25α complex in the presence or absence of AMP or ADP (both 300 µM), as described in the Materials and methods section. After the incubation aliquots were withdrawn for (**A**) AMPK assay or (**B**) Western blots. For (**A**), results show mean ± SEM (*n* = 3); mean values significantly different from those obtained with WT AMPK are shown (****P < 0.0001); ns, not significant. For (**B**), due to a limit on the number of lanes that could be run on one gel, single replicates from the triplicate assays were analysed.

**Figure 6.**
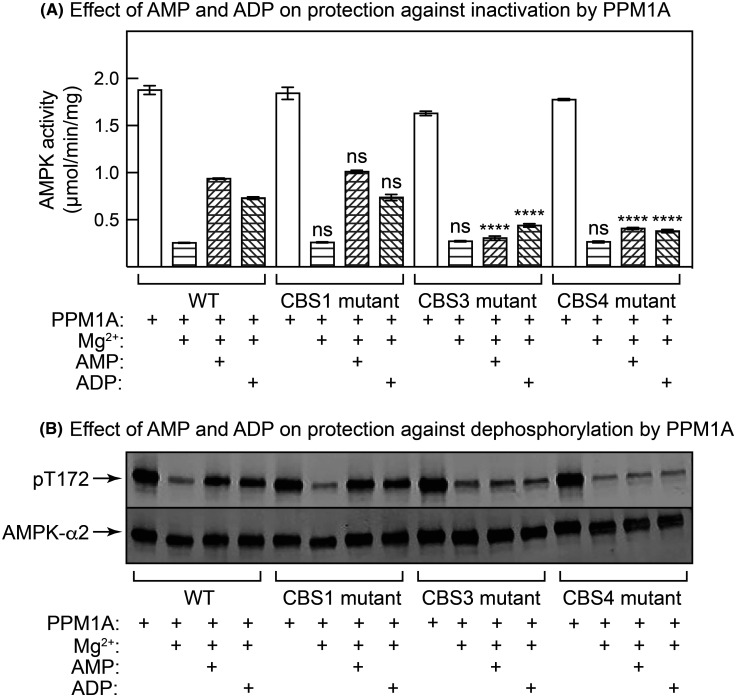
Effects of AMP or ADP on AMPK inactivation and Thr172 dephosphorylation by PP2Cα (PPM1A). Human α2β2γ1 complexes (WT, or with L129D/V130D, V276G/L277G or I312G mutations affecting CBS1, CBS3 or CBS4 respectively), all phosphorylated on Thr172, were incubated with a fixed concentration of PP2Cα (PPM1A) in the presence or absence of AMP or ADP (both 300 µM) as described in the Materials and methods section. After the incubation, aliquots were withdrawn for (**A**) AMPK assay or (**B**) Western blots. For (**A**), results show mean ± SEM (*n* = 3); mean values significantly different from those obtained with WT AMPK are shown (****P < 0.0001); ns, not significant. For (**B**), due to a limit on the number of lanes that could be run on one gel, single replicates from the triplicate assays were analysed.

The relative roles of the three CBS sites in the other two mechanisms of activation of AMPK by AMP or ADP, i.e. promotion of Thr172 phosphorylation by LKB1, and protection against Thr172 dephosphorylation by protein phosphatases, have not been examined previously. As expected, incubation of the α2β2γ1 AMPK complex with a fixed, limiting amount of purified LKB1:STRADα:MO25α complex in the presence of 200 µM Mg.ATP^2−^ caused a large degree of activation (Figure 5A) and phosphorylation (Figure 5B) of AMPK (unphosphorylated α2β2γ1 complex), which were stimulated more than 2-fold by 300 µM AMP and 1.6-fold by 300 µM ADP. These effects of AMP or ADP were unaffected by mutations in the CBS1 nucleotide binding site, but were abolished by mutations in the CBS3 or CBS4 sites, demonstrating the critical role of the latter sites in these effects. Also as expected, incubation with a fixed, limiting amount of purified PPM1A (PP2C-α) in the absence of ATP caused a large inactivation (Figure 6A) and dephosphorylation (Figure 6B) of the phosphorylated α2β2γ1 complex, effects that were greatly reduced by the presence of 300 µM AMP or ADP. Once again, these effects of AMP or ADP were unaffected by mutations in the CBS1 binding site but were almost completely abolished by mutations in the CBS3 or CBS4 sites, demonstrating the critical roles of the latter two sites in the effects of adenine nucleotides on Thr172 dephosphorylation.

## Discussion

Our results reveal that binding of ADP to the AMPK-γ subunit does indeed cause allosteric activation of the heterotrimeric complex. Very similar results were obtained with native rat liver AMPK and with a recombinant human α2β2γ1 complex that had been phosphorylated on Thr172 by CaMKK2, despite different species of origin, subunit isoform composition and N-myristoylation status, and the presence of a polyhistidine tag on the latter. The effect of ADP was most likely missed in previous studies [6,7] because assays had been performed under the standard assay conditions of 0.2 mM ATP, under which conditions we did not observe allosteric activation by ADP either. Although a low ATP concentration is used in the standard assay because it allows use of a higher specific radioactivity of labelled ATP, the concentrations of ATP in intact cells are thought to be in the mM range, with 5 mM being a good approximation to the likely concentration in unstressed cells [21].

The allosteric activation by ADP that we observed cannot be explained either by contamination of our ADP preparation by AMP or by generation of AMP from ADP during the assay [4,20], because inclusion in the assays of the 5′-nucleotidase CD73 (which converts AMP to adenosine but is completely inactive against ADP) completely prevented allosteric activation by AMP, but did not abolish activation by ADP.

Although ADP does therefore cause allosteric activation of AMPK at normal cellular ATP concentrations, this may not have much relevance in physiological settings. For rat liver AMPK, the EC_50_ for allosteric activation by ADP at 5 mM ATP (220 µM) was only 18-fold lower than the IC_50_ for inhibition at the catalytic site (4 mM), and with the human α2β2γ1 complex they were even closer together (0.8 and 1.2 mM). Thus, the activating and inhibitory phases for ADP are overlapping. This means that inhibition starts to occur at concentrations only slightly higher than those that cause activation, and the maximal extents of activation are therefore small (≈1.5-fold). In contrast, in the case of AMP when measured with 5 mM ATP in the assay, the EC_50_ for allosteric activation (36 µM) of rat liver AMPK was almost 500-fold lower than the IC_50_ for inhibition at the catalytic site (17 mM). Thus, in the case of AMP the activating and inhibitory phases are well separated, and the maximal extent of activation (3-fold) is much larger than that for ADP. Moreover, we have previously estimated that, in cells treated with the mitochondrial inhibitor berberine, AMP rises from 40 to 240 µM while ADP rises from 430 to 980 µM [4]. These estimated changes are within the rising phases of the bell-shaped curves for AMP (Figures 1C and 4E) and would be expected to cause a large stimulation, whereas they lie near the top of the bell-shaped curves for ADP and would be expected to have little effect.

 Our results for allosteric activation by AMP at 200 µM ATP are consistent with those in more limited previous studies where hydrophobic residues [12] or conserved aspartate residues [18] involved in binding AMP in CBS1, CBS3 and CBS4 were mutated, and allosteric activation was measured at a single concentration of AMP. In those studies, mutation of CBS1 had only modest effects on AMP activation, whereas mutation of CBS3 or CBS4 had more drastic effects.

 While the effects of CBS mutations on allosteric activation by AMP had been previously studied [12,18], their effects on the other two mechanisms of AMPK activation, i.e. promotion of Thr172 phosphorylation and protection against Thr172 dephosphorylation, had not. By mutating hydrophobic residues in AMPK-γ1 involved in binding the adenine moieties of AMP, ADP and ATP in the CBS sites [12,14], we assessed the importance of the three nucleotide-binding sites on all three mechanisms of activation by AMP and ADP. Melcher's group [14,17] have used various approaches, including structural biology, to support the idea that CBS3 is the high affinity binding site for AMP where it binds in competition with ATP, with displacement of ATP by AMP at this site causing a major conformational change [17] that triggers activating mechanisms. They also proposed that the non-exchangeable binding of AMP at the CBS4 site increases the binding affinity for AMP (relative to ADP and ATP) at the neighbouring CBS3 site, most likely by repositioning the side chains of His298 and Arg299 (human γ1 numbering), which are present in CBS4 but bind the phosphate and adenine moieties of AMP bound to CBS3. From Melcher's studies [14], the role of the CBS1 site, if any, is much less clear. Our results with γ1 mutations affecting CBS1, CBS3 and CBS4 are broadly in line with Melcher's proposals. Thus, when the assays were conducted at low ATP concentration (200 µM), mutations affecting nucleotide binding at the CBS1 site had little or no effect, mutations affecting CBS3 almost abolished allosteric activation by AMP, while mutations affecting CBS4 had little effect on maximal activation by AMP but increased the EC_50_ 40-fold from 1.1 to 45 µM. Thus, while the CBS3 site is essential and the CBS1 site dispensable for allosteric activation by AMP, at this ATP concentration (when ADP has no effect), our results are consistent with the suggestion [14] that binding of AMP at the non-exchangeable CBS4 site increases the affinity for AMP binding at the crucial, neighbouring CBS3 site.

 Our results were somewhat different when the assays were carried out at the higher, more physiological ATP concentration of 5 mM. In this case, mutations affecting CBS1 reduced the maximal activation by AMP from 3.0- to 2.3-fold without affecting the EC_50_, while mutations affecting CBS3 and CBS4 reduced the maximal activation to 2.0- to 2.5-fold but also increased the EC_50_ values by 10- to 20-fold, indicating a large drop in sensitivity to AMP. In the case of ADP, mutations affecting any of the three CBS sites essentially abolished allosteric activation. Thus, binding of nucleotides at the CBS1 site appears to become more important as the ATP concentration is increased.

The shape of the curves in Figures 1C and 4E, showing allosteric activation by ADP at 5 mM ATP for the rat liver and human α2β2γ1 complexes, are remarkably similar to our previous results (Figure 6C in ref. [8]) in which we measured the effect of ADP on activation of FLAG-γ1-containing AMPK complexes expressed in HEK-293 cells by the upstream kinase LKB1. At concentrations from 300 µM to 3 mM, ADP caused a modest (<2-fold) stimulation of activation and then at slightly higher concentrations caused a marked inhibition, presumably because ADP competes with ATP for binding at the catalytic site of LKB1. Thus, the effect of ADP on promotion of Thr172 phosphorylation by LKB1 is subject to the same limitations as its allosteric effects, i.e. that at concentrations of ADP only slightly above those that cause activation, the nucleotide starts to inhibit.

Our results for the effects of AMP and ADP on promotion of phosphorylation at Thr172 by LKB1 (carried out at 200 µM ATP), and protection against Thr172 dephosphorylation by PPM1A (carried out in the absence of ATP) once again suggest that binding of AMP at CBS3 and CBS4 is of crucial importance, while the CBS1 site is less important. In these experiments, mutations affecting CBS1 had no effect, whereas mutations affecting AMP binding to either CBS3 or CBS4 abolished the effects. Thus, all three mechanisms of activation of AMPK by AMP appear to be triggered by binding at the same sites.

In conclusion, ADP can cause allosteric activation of the AMPK complex under certain conditions in cell-free assays. We cannot completely rule out the possibility that any of the three activation mechanisms triggered by ADP might play a minor role *in vivo*. However, our findings that ADP has inhibitory effects at concentrations that overlap with its activating effects suggests that it is unlikely to be as important as AMP as a physiological regulator of AMPK. In the case of AMP, the concentrations causing activation and those causing inhibition are well separated and not overlapping, and we would thus argue that AMP is the crucial activator of the AMPK system *in vivo*. Our results with mutations affecting binding of nucleotides at the CBS3 and CBS4 sites support the proposal of Melcher [14] that CBS3 is the crucial high-affinity binding site for AMP. Displacement of ATP by AMP at this site causes a large conformational change [17] that now appears to trigger all three mechanisms of activation (promotion of Thr172 phosphorylation, inhibition of Thr172 dephosphorylation, and allosteric activation). The role of nucleotide binding at CBS1 remains less clear, although it appears to become more important at higher ATP concentrations, when its disruption reduces allosteric activation by both AMP and ADP. Interestingly, His151 in CBS1 interacts with the phosphate groups of AMP bound in both the CBS1 and CBS4 sites [14], potentially explaining how nucleotide binding at CBS1 can affect the more crucial CBS3 and CBS4 sites. Further structural analyses may be required to fully elucidate the complex mechanisms of nucleotide sensing displayed by AMPK complexes.

## Materials and methods

### Antibodies, recombinant proteins and other materials

Sources of antibodies were as described: pThr172 (AMPK-α: Cell Signaling Technologies, Danvers, MA, U.S.A., Cat# 2535), AMPK-α2 [22]. Rat liver AMPK was purified as described previously [4]. Plasmid encoding human (His)_6_-tagged AMPK (α2β2γ1, bacterially expressed) generated as in [23], was a gift from AstraZeneca, Cambridge, U.K. The L129D/V130D (CBS1), V276G/L277G (CBS3) and I312G (CBS4) mutations in the α2β2γ1 complex [12,14] were generated using the Quikchange II site-directed mutagenesis kit (Agilent Technologies) and confirmed by DNA sequencing. Human GST-tagged α2 kinase domain (1–310) [24], LKB1:STRAD-α:MO25-α complexes [25], GST-tagged CaMKK2 [26], and PPM1A (PP2Cα) [27] were produced as described. Bacterially expressed heterotrimers were activated by phosphorylation of Thr172 by GST-tagged CaMKK2 in the presence of ATP, and CaMKK2 was subsequently removed by passage through glutathione-Sepharose [28]. Human GST-tagged α2 kinase domain was activated in a similar manner except that human (His)_6_-tagged LKB1:STRAD-α:MO25-α complex was used and removed after activation via passage through a His-Trap column. Recombinant human 5′-nucleotidase, CD73, Cat # 5795-EN-01, was from R&D systems (Minneapolis, MN, U.S.A.).

### AMPK assays and assays of phosphorylation and dephosphorylation of AMPK

AMPK assays measured the transfer of radioactivity from [γ-^33^P]ATP to a peptide substrate, which was separated from unreacted ATP by binding to P81 paper. This assay was described previously [29] except that we used [γ-^33^P] rather than [γ-^32^P]ATP. In some assays (specified in Figure legends) the concentration of ATP was increased from 0.2 to 1 or 5 mM ATP, with MgCl_2_ concentrations maintained at a constant 4.8 mM excess above the concentration of ATP [19]. Where allosteric activation by AMP or ADP was being studied, we used the *SAMS* peptide as substrate. For all other assays the *AMARA* peptide was used [30].

For assays of AMPK phosphorylation by the LKB1 complex, human α2β2γ1 complex (250 ng of WT or CBS mutant, as indicated) was incubated in a shaking incubator at 30°C for 12 min in Hepes buffer (50 mM Na Hepes, pH 7.4, 150 mM NaCl, 1 mM dithiothreitol, 0.02% (w/v) Brij-35) with 200 µM ATP and 5 mM MgCl_2,_ with or without a limiting amount of LKB1:STRAD-α:MO25-α complex (0.05 µg) in the presence or absence of either 300 µM AMP or 300 µM ADP (total volume 25 µl). After 12 min, aliquots (equivalent to 50 ng of heterotrimeric complex) were removed for AMPK activity assay using the *AMARA* peptide as substrate and in the presence of 200 µM AMP, or Western blot analysis.

For assays of dephosphorylation by PPM1A (PP2Cα), human AMPK α2β2γ1 complex (2.5 mg of WT or CBS mutant as indicated; 25 µl final volume) was incubated in a shaking incubator at 30°C for 12 min in Hepes buffer with 50 mM MgCl_2_ and sufficient PPM1A to yield ∼80–90% inactivation in the absence of AMP or ADP (total volume 25 µl). The reaction was terminated by addition of 350 µl of Hepes buffer and aliquots (equivalent to 50 ng of heterotrimeric complex) were removed for AMPK activity assay using the *AMARA* peptide as substrate in the presence of 200 µm AMP, or for Western blot analysis.

### SDS–PAGE and other analytical procedures

SDS–PAGE was performed using precast NuPAGE Bis-Tris 4–12% gradient polyacrylamide gels in the MOPS buffer system (ThermoFisher Scientific, Waltham, MA, U.S.A.). Proteins were transferred to nitrocellulose membranes using the iBlot 2 system (ThermoFisher Scientific). Membranes were blocked for 1 h in Li-Cor Odyssey blocking buffer and then probed with the appropriate antibody (0.1 mg/ml) overnight at 4°C. Detection was performed using a secondary antibody coupled to IR 680 or IR 800 dye and the membranes scanned using the LI-COR Odyssey IR imager. Protein concentrations were determined by Coomassie Blue binding with bovine serum albumin as a standard [31].

### Statistical analysis

Statistical significance was tested using GraphPad Prism 10 for MacOS, using tests for significance specified in Figure legends. The Holm-Sidak method was used to correct for multiple comparisons. Curve fitting was also carried out using GraphPad Prism 10 using equations specified in Figure legends.

## Data Availability

With the exception of pilot studies, all data relevant to this paper are included in the manuscript.

## Open Access

Open access for this article was enabled by the participation of University of Dundee in an all-inclusive *Read & Publish* agreement with Portland Press and the Biochemical Society under a transformative agreement with JISC.

## Abbreviations

AMPK: AMP-activated protein kinase
CBS: cystathione β-synthase
CD73: cluster of differentiation-73
EC_50_: concentration causing half-maximal activation
GST: glutathione *S*-transferase
IC_50_: concentration causing half-maximal inhibition
LKB1: liver kinase-B1
WT: wild type
