# Supplementary material for: AMP-activated protein kinase can be allosterically activated by ADP but AMP remains the key activating ligand
Source: Biochem J. 2024 Apr 22;481(8):587–99. doi: 10.1042/BCJ20240082 (PMC11088877; doi:10.1042/BCJ20240082)
Supplement: Supplementary Material [file BCJ-481-587-s1.pdf]

Hawley et al Figure S1

A) Coomassie Blue staining of purified rat liver and human α2β2γ1 complexes

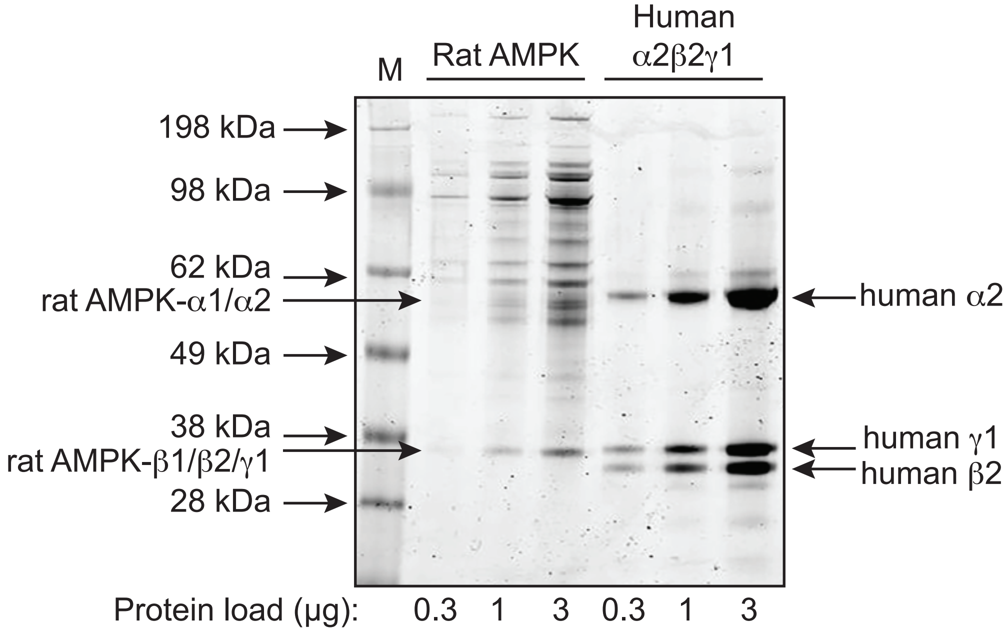

B) Western blotting of purified rat liver (R) and human α2β2γ1 (H) complexes

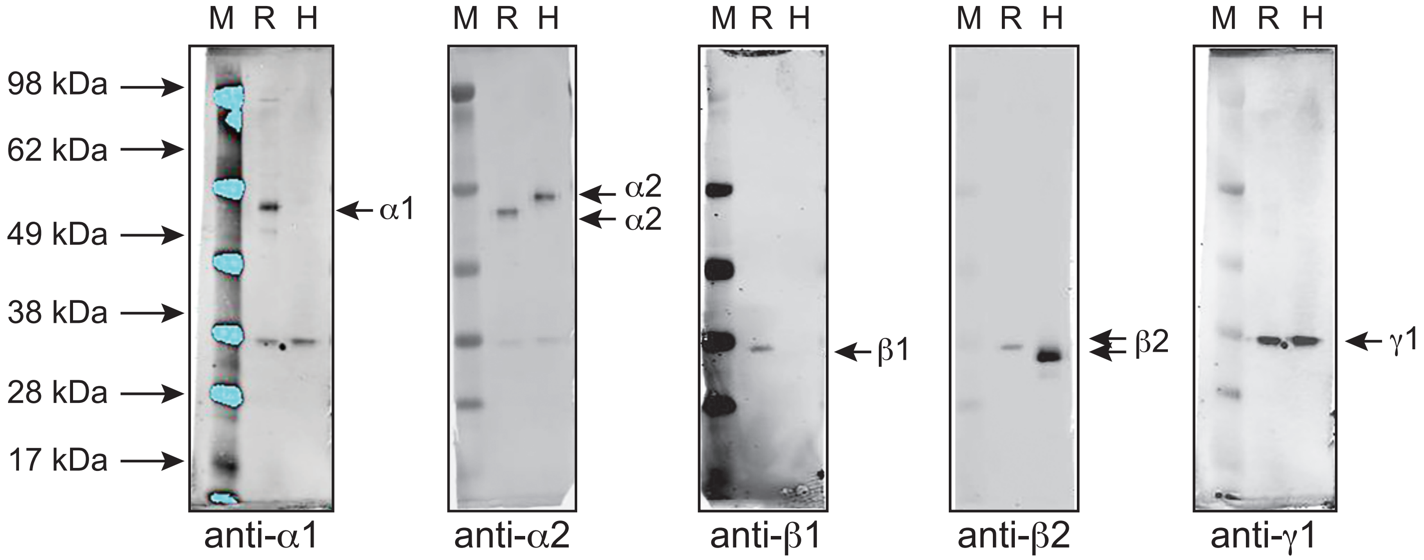

Figure S1: Analysis by Coomassie Blue staining (A) and Western blotting (B) of the purified preparations of rat liver and human (α2β2γ1) AMPK complexes used in this study. The markers used (lane M) were SeeBlue Plus2 Prestained Protein Standards (ThermoFisher Scientific), and the molecular masses are those given by the manufacturer for the gel system used. The AMPK subunit isoforms labelled in (A) were identified by reference to the Western blots in (B). Note from the Western blots in (B) that rat β1, β2 and γ1 co-migrate and are therefore not resolved in the Coomassie Blue-stained gel in (A).

Hawley et al Figure S2

A) γ1 mutants co-purify with α2 and β2 as stable heterotrimers

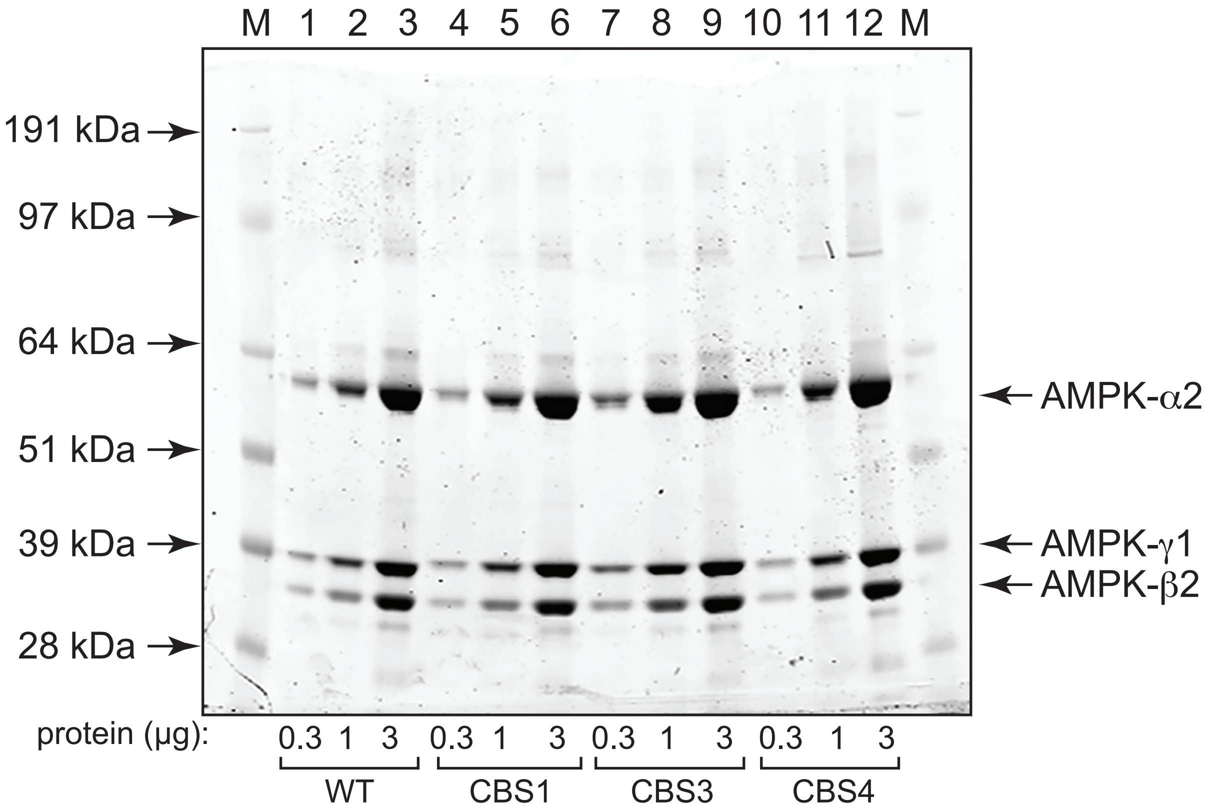

B) γ1 mutants all activated by CaMKK2

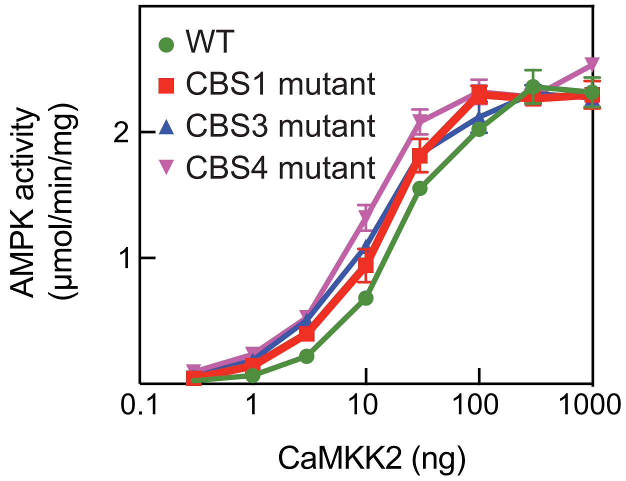

C) γ1 mutants all activated by LKB1

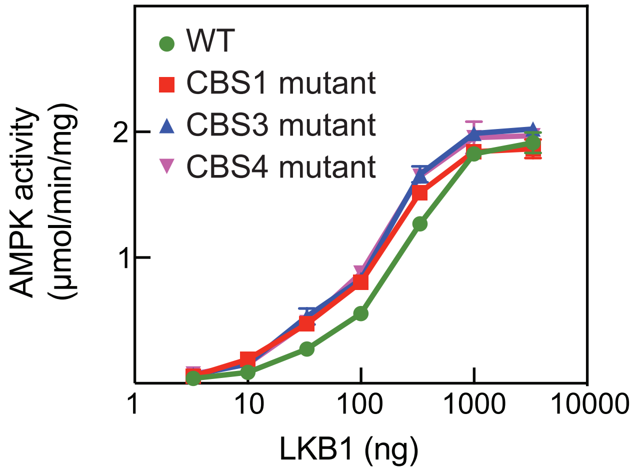

Figure S2: (A) Analysis by SDS-PAGE and Coomassie Blue staining of purified bacterially expressed human α2β2γ1 complexes, either WT or with mutations affecting CBS1, CBS3 or CBS4; (B) activation of WT or CBS mutants using increasing concentrations of CaMKK2; (C) activation of WT or CBS mutants using increasing concentrations of LKB1:STRADα:MO25α complex. For (A), the complexes had been purified using the $(His)_6$ tag on the α2 subunit; the gel shows that stable αβγ complexes are formed with the WT and each mutant. (B) and (C) show that the mutations do not affect phosphorylation and activation by either upstream kinase.

Fig. 5B/6B: uncropped blots (promotion/protection of phosphorylation/dephosphorylation)

Fig. 5B:

pT172 →

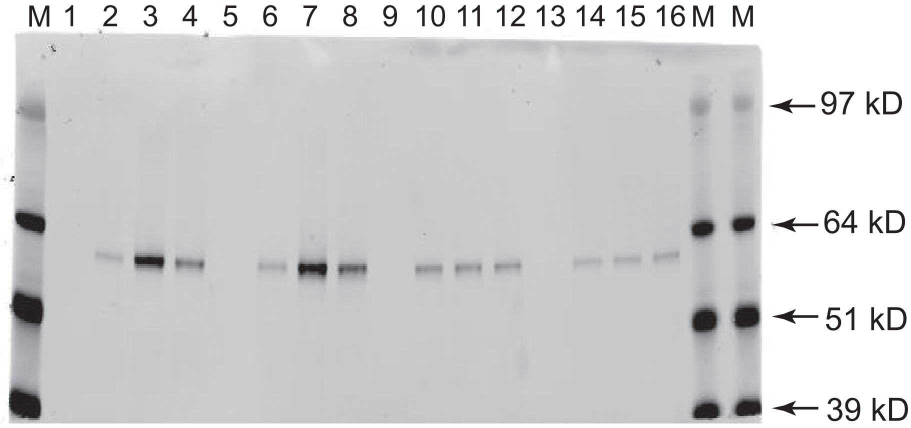

Fig. 5B:

AMPK-α2 →

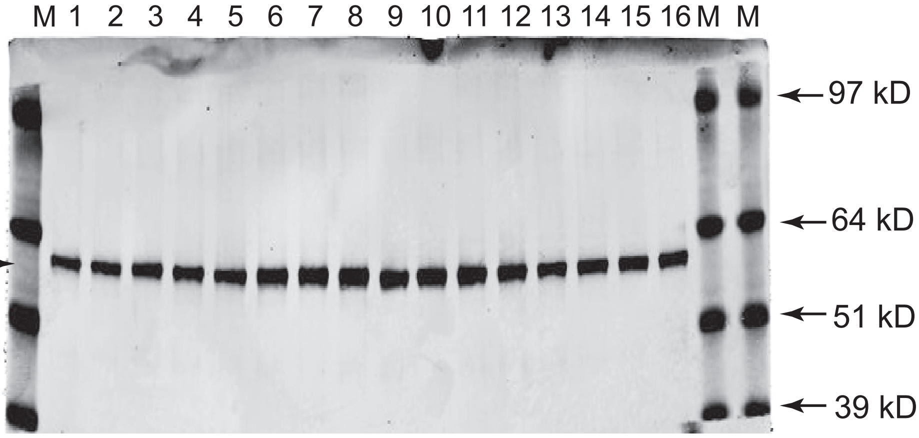

Fig. 6B:

pT172 →

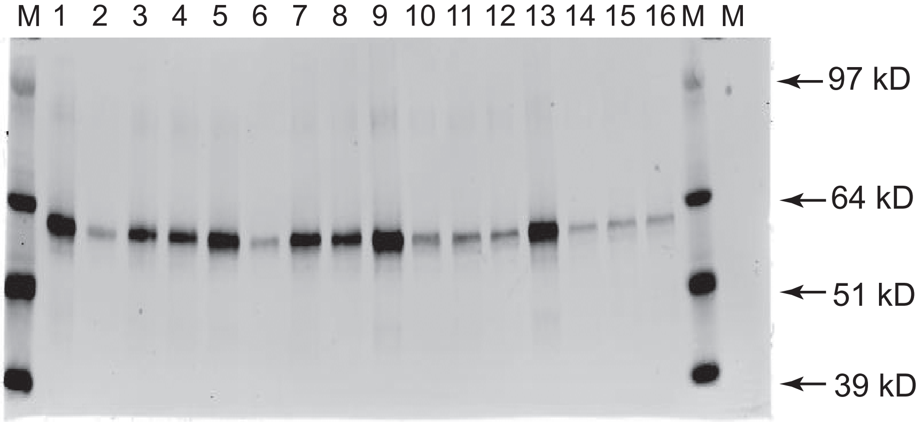

Fig. 6B:

AMPK-α2 →

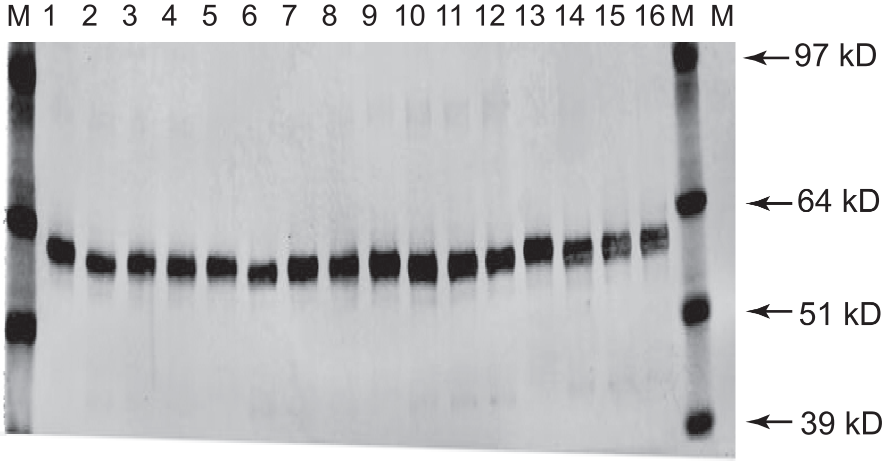

NOTES:

- the molecular mass markers used (in lanes designated M) were SeeBlue Plus2 prestained protein standards (Cat. no. LC5925, ThermoFisher Scientific)
- the molecular masses given for the markers are those quoted by the manufacturer's for the gel system used

Enzyme	Activator	ATP (mM)	Basal ± SD (CI) (nmol/min/mg)	Activation (fold)	EC_{50} (μM)	IC_{50} (mM)	IC_{50}/EC_{50}
Rat liver	AMP	0.2	121 ± 4	2.9 (2.8-3.0)	6.4 (5.4-7.5)	1.7 (1.6-2.0)	270
Rat liver	AMP	1.0	129 ± 4	2.8 (2.7-2.9)	14 (12-16)	6.9 (6.1-7.8)	490
Rat liver	AMP	5.0	96 ± 4	3.7 (3.5-3.8)	36 (28-46)	17 (13-23)	470
Rat liver	ADP	0.2	161 ± 7	none*	-	0.40 (0.36-0.44)	-
Rat liver	ADP	1.0	147 ± 7	2.4 (1.8-??)	122 (65-??)	0.69 (0.61-0.78)	5.7
Rat liver	ADP	5.0	123 ± 1	2.0 (1.7-2.8)	220 (120-440)	4.0 (2.7-5.4)	18
α2-KD	AMP	0.2	1020 (990-1040)	none*	-	2.2 (1.8-2.7)	-
α2-KD	AMP	1.0	2410 (2360-2460)	none*	-	5.4 (4.5-6.5)	-
α2-KD	AMP	5.0	2300 (2260-2340)	none*	-	13.4 (11.1-16.4)	-
α2-KD	ADP	0.2	1180 (1150-1210)	none*	-	0.67 (0.59-0.77)	-
α2-KD	ADP	1.0	2600 (2550-2660)	none*	-	1.6 (1.4-1.9)	-
α2-KD	ADP	5.0	2630 (2570-2690)	none*	-	3.5 (3.0-4.1)	-
α2β2γ1 WT	AMP	0.2	1558 ± 36	2.6 (2.5-2.6)	1.0 (0.8-1.2)	2.6 (2.3-2.9)	2600
α2β2γ1 WT	AMP	5.0	1363 ± 40	3.0 (2.9-3.1)	19 (15-24)	52 (41-68)	2700
α2β2γ1 WT	ADP	0.2	1448 ± 15	none*	-	2.6 (??-??)	-
α2β2γ1 WT	ADP	5.0	1334 ± 81	9.9 (2.8-??)	785 (321-??)	1.2 (??-2.5)	1.5
α2β2γ1 CBS1	AMP	0.2	1590 ± 47	2.4 (2.3-2.4)	1.6 (1.3-1.9)	3.4 (3.1-3.8)	2100
α2β2γ1 CBS1	AMP	5.0	2219 ± 154	3.0 (2.9-3.1)	19 (15-24)	52 (41-68)	2700
α2β2γ1 CBS1	ADP	0.2	1879 ± 67	none*	-	1.0 (0.45-??)	-
α2β2γ1 CBS1	ADP	5.0	1880 ± 106	none*	-	1.2 (??-2.5)	-
α2β2γ1 CBS3	AMP	0.2	1402 ± 0	1.2 (1.1-1.2)	2.9 (0.7-5.1)	6.1 (5.0-7.4)	2100
α2β2γ1 CBS3	AMP	5.0	1774 ± 95	2.3 (2.1-2.7)	370 (220-650)	40 (27-60)	110
α2β2γ1 CBS3	ADP	0.2	1629 ± 43	none*	-	0.5 (0.3-??)	-
α2β2γ1 CBS3	ADP	5.0	1363 ± 14	-	-	7.6 (4.5-??)	-
α2β2γ1 CBS4	AMP	0.2	1539 ± 8	2.1 (2.0-2.1)	38 (30-49)	3.3 (2.9-3.8)	87
α2β2γ1 CBS4	AMP	5.0	2047 ± 59	2.1 (1.9-2.4)	280 (160-500)	28 (20-41)	100
α2β2γ1 CBS4	ADP	0.2	1766 ± 23	none*	-	0.78 (0.35-??)	-
α2β2γ1 CBS4	ADP	5.0	1459 ± 69	none*	-	31 (??-??)	-

Table S1: Summary of parameters estimated from data in Figures 1, 3 and 4. These parameters were used to draw the curves in those Figures, using the equations given in Figure legends. Figures in parentheses indicate 95% confidence intervals for that parameter estimated by curve fitting, while the figures after "±" symbols are standard deviations calculated from the individual basal activities. "??" indicates that that confidence interval could not be reliably determined. The entry "none*" indicates that the activation was not significant and parameters relevant to activation could not be reliably determined.
